# *MAGEA11* as a STAD Prognostic Biomarker Associated with Immune Infiltration

**DOI:** 10.3390/diagnostics12102506

**Published:** 2022-10-16

**Authors:** Chen Xiao, Linhui Yang, Liangzi Jin, Faqin Zhang, Jingbo Liu, Chunyu Yu, Lei Tao, Changfu Li

**Affiliations:** 1 Department of Gastroenterology, Fuzhou Second Hospital, The Third Clinical Medical College, Fujian Medical University, Fuzhou 350001, China; 2 Institute of Medical Biology, Chinese Academy of Medical Sciences, Peking Union Medical College, Kunming 650000, China; 3 Department of Pathology, Daqing Longnan Hospital Affiliated with Qiqihar Medical University, Daqing 163000, China; 4 Department of Orthopedic Surgery, Spine Center, Changzheng Hospital, Naval Medical University, Shanghai 200000, China; 5 Department of Digestive Internal Medicine, Daqing Longnan Hospital, The Fifth Affiliated Hospital of Qiqihar Medical College, Daqing 163000, China

**Keywords:** gastric cancer, *MAGEA11*, biomarker, prognosis, immune infiltration

## Abstract

Expression of MAGE family member A11 (*MAGEA11*) is upregulated in different tumors. However, in gastric cancer, the prognostic significance of *MAGEA11* and its relationship with immune infiltration remain largely unknown. The expression of *MAGEA11* in pan-cancer and the receiver operating characteristic (ROC) and survival impact of gastric cancer were evaluated by The Cancer Genome Atlas (TCGA). Whether *MAGEA11* was an independent risk factor was assessed by Cox analysis. Nomograms were constructed from *MAGEA11* and clinical variables. Gene functional pathway enrichment was obtained based on *MAGEA11* differential analysis. The relationship between *MAGEA11* and immune infiltration was determined by the Tumor Immunity Estimation Resource (TIMER) and the Tumor Immune System Interaction Database (TISIDB). Finally, *MAGEA11*-sensitive drugs were predicted based on the CellMiner database. The results showed that the expression of *MAGEA11* mRNA in gastric cancer tissues was significantly higher than that in normal tissues. The ROC curve indicated an AUC value of 0.667. Survival analysis showed that patients with high *MAGEA11* had poor prognosis (HR = 1.43, *p* = 0.034). In correlation analysis, *MAGEA11* mRNA expression was found to be associated with tumor purity and immune invasion. Finally, drug sensitivity analysis found that the expression of *MAGEA11* was correlated with seven drugs. Our study found that upregulated *MAGEA11* in gastric cancer was significantly associated with lower survival and invasion by immune infiltration. It is suggested that *MAGEA11* may be a potential biomarker and immunotherapy target for gastric cancer.

## 1. Introduction

Gastric cancer, the world’s fifth most common cancer type, was diagnosed with approximately 1.1 million new cases in 2020 (5.6% of all cancer cases) [1]. Gastric cancer is also one of the most lethal malignancies, with a five-year survival rate of approximately 20% [2]. Among them, stomach adenocarcinoma (STAD) is the main pathological type of gastric cancer. Multiple clinical factors, such as age, tumor size, histological grade, lymphatic invasion, and number of lymph node metastases, have been shown in studies to affect the prognosis of gastric cancer [3,4]. Because of the complexity of gastric cancer incidence and tumor heterogeneity, despite the discovery of many prognostic markers, prediction efficiency remains insufficient [5,6,7,8]. Therefore, gastric cancer patients require new biomarkers for improved treatment and prognosis.

MAGE is a family of cancer antigens; members of the MAGEA family are involved in the regulation of apoptosis, cell cycle progression, and cell differentiation and proliferation [9], usually detected in cancer cells; and they can be found in lung cancer, breast cancer, and urothelial cancer [10]. It is highly expressed in various cancers such as malignant tumor, oral squamous cell carcinoma, esophageal carcinoma, hematopoietic malignant tumor, prostate cancer, and skin melanoma [11,12,13,14,15,16,17,18]. In healthy human reproductive tract tissue, melanoma antigen family A11 (*MAGEA11*) is a low-abundance, primate-specific steroid receptor coregulator that is crucial for tumorigenesis [19]. Currently, it is believed that *MAGEA11* plays a role in regulating the androgen receptor signaling network. Through a chemical mechanism, it can boost the androgen receptor’s transcriptional activity [20,21]. *MAGEA11* also acts as a human progesterone receptor-specific coregulator by interacting with the progesterone receptor [22]. *MAGEA11* increases steroid receptor transcriptional activity in primates by interacting with p300 histone acetyltransferase [23] and p160 steroid receptor coactivator [24]. It has been reported that *MAGEA11* also affects the cell cycle [12]. Studies have found that prostate cancer has abnormally high levels of *MAGEA11* and high androgen receptor protein expression, and increased *MAGEA11* expression is directly related to the progression of prostate cancer [23]. Recently, scientific interest in *MAGEA11* has increased, but its association with gastric cancer is limited.

In this research, we investigated the expression of *MAGEA11* in gastric cancer and used bioinformatics analysis to evaluate its correlation with the prognosis and immune infiltration of gastric cancer patients and the sensitivity to different chemotherapeutic drugs. Our study showed that upregulation of *MAGEA11* in gastric cancer was significantly associated with decreased survival and invasion by immune infiltration.

## 2. Materials and Methods

### 2.1. Transcription Data

Visit The Cancer Genome Atlas (TCGA) website [25] to download the transcription and expression data of *MAGEA11* and the associated clinical data. From 33 registered cancers, more than 5 samples were chosen for examination. Finally, log2 transformation and conversion of the workflow type FPKM RNA-seq gene expression data into TPM format were performed in preparation for further research. At the same time, we supplemented the sequencing data of normal gastric tissue included in the GTEx database and applied the same normalization as TCGA. Moreover, the GEO datasets (GSE54129, GSE84437) were also used for further validation. Ethics committee approval was not required for this study as all data were downloaded from TCGA, GTEx, and GEO.

### 2.2. RNA Sequencing Data of MAGEA11 in Gastric Cancer

We conducted a joint analysis of the sequencing data through the Xiantao platform (https://www.xiantao.love/products (accessed on 10 June 2022)). Therefore, 375 gastric cancers and 32 nearby normal tissues were preserved in TCGA, while 174 normal gastric tissues were preserved in GTEx. The chosen samples included information on the *MAGEA11* gene expression as well as clinically relevant data, such as age, T stage, N stage, M stage, etc. 

### 2.3. The Subcellular Distribution of MAGEA11

Using the HPA database (https://www.proteinatlas.org/ (accessed on 10 June 2022)), the subcellular distribution of the protein was investigated by immunofluorescence and confocal microscopy in up to cell lines.

### 2.4. Gene Enrichment Analysis

GSEA [26] was used to find meaningful biological features in the high and low expression *MAGEA11* population in the STAD cohort. Using the “ggplot2”, “enrichPlot”, and “clusterProfiler” R package, GSEA, and functional pathway analysis were carried out between the two groups. Filter conditions include the following: adjusted *p*-value < 0.05, richness of normalized scores (|NES|) < 1, and false discovery rate (FDR) < 0.25 were considered significant differences. Gene Ontology (GO) is a widely used method in bioinformatics to explore which functions of gene sets are significant. The analysis of cellular component (CC), biological process (BP), and molecular function (MF) can be performed according to candidate genes. Kyoto Encyclopedia of Genes and Genomes (KEGG) is a novel approach to understanding advanced functions and biological systems from the level of molecular information, especially by genome sequencing and other high-throughput experimental techniques generated from large molecular datasets. GO and KEGG were analyzed for enrichment through the “clusterProfiler” package, and “org.Hs.eg.db” was applied for ID transformation, and the “ggplot2” package was used for data visualization. A filter condition of *p* < 0.05 was set.

### 2.5. Construction and Evaluation of Nomograms

Based on the results of the multivariate analysis, individual predictive nomograms [27] for 1-, 3-, and 5-year survival probability were constructed. The “RMS” ”survival” package was used to generate nomograms with important clinical characteristics and calibration plots. The calibration curve was used to estimate its predictive power.

### 2.6. Protein–Protein Interaction (PPI) Network and Functional Enrichment Analysis

An online database called STRING [28] (https://www.string-db.org/ (accessed on 10 June 2022)) was used to look up interacting genes. The data of protein interaction are mainly obtained through experiments, text mining, database mining, gene adjacency, co-expression, and other methods. A PPI network [29] was created in this study by using STRING to look for co-expressed genes and a setting of 0.4 for the interaction score. The enrichment analysis of functional pathways of interacting genes also used GO and KEGG analysis.

### 2.7. Gene Alteration Analysis

Through the cBioPortal database (https://www.cbioportal.org/ (accessed on 10 June 2022)), the genetic alteration profile of *MAGEA11* was discovered. In the Cancer Type Summary module, findings for altered frequency, mutation type, and copy number alterations (CNAs) were seen across all TCGA tumors. The “mutation” module’s three-dimensional structure can display the *MAGEA11* mutation site information. Somatic copy number changes (sCNAs) represent an important class of mutations in the cancer genome. Using the Tumor Immunity Estimation Resource (TIMER) database (https://cistrome.shinyapps.io/timer/ (accessed on 10 June 2022)), immune infiltration distribution can be compared by sCNA status. Using copy number segmentation profiles, GISTIC2. 0 (25) calculates the sCNA information at the gene level, including “deep deletion”, “arm-level deletion”, “diploid/normal”, “arm-level gain”, and “high amplification”. The violin plot depicted the distribution of immune infiltration among the various sCNA states of the gene.

### 2.8. Immune Microenvironment Correlation Analysis

The relationship between *MAGEA11* and immune checkpoints was analyzed by the TIMER database. The Tumor Immune System Interaction Database (TISIDB) (http://cis.hku.hk/TISIDB/ (accessed on 10 June 2022)) was used to determine the expression of *MAGEA11* and tumor-infiltrating lymphocytes (TILs) in human cancers. Genomic variation analysis and gene expression profiles were used to infer the relative abundance of TILs. *MAGEA11* and TILs’ correlation was evaluated using Spearman’s test. The relative abundance threshold was set to *p* value < 0.05.

### 2.9. Drug Sensitivity Analysis

The National Cancer Institute (NCI) Cancer Research Center’s list of 60 cancer cells is compiled in the CellMiner database. For testing anticancer drugs, the NCI-60 cell line is currently the most frequently used cancer cell sample group. Through correlation analysis, this study examined the relationship between genes and common antitumor drug sensitivity, it downloaded NCI-60 drug sensitivity data and RNA-seq gene expression data.

### 2.10. Statistical Analysis

R (V4.1.2) was used to perform all statistical analyses, and Cox regression analysis was performed through the “survival” package to identify independent prognostic factors for overall survival. To distinguish between normal tissue and gastric cancer tissue, paired T tests or Mann–Whitney U tests were applied. ROC curves were used to detect *MAGEA11* cutoffs [30,31] using the pROC package.

## 3. Results

### 3.1. Expression of MAGEA11 in Tumors

**Figure 1:**
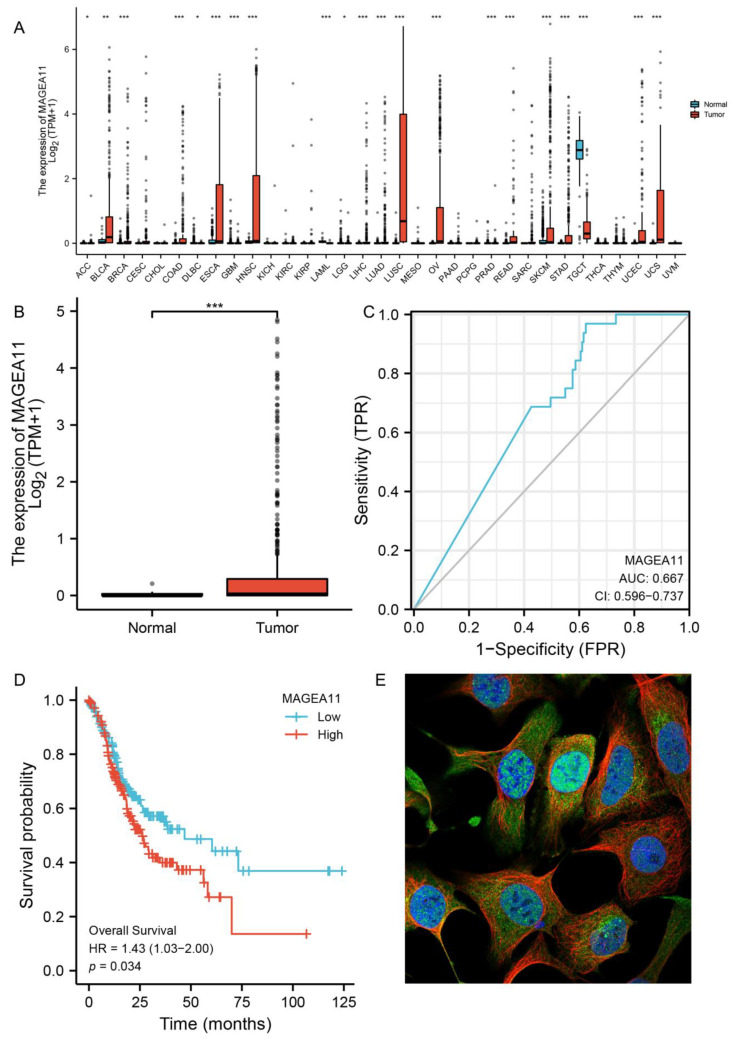
*MAGEA11* expression and prognosis. (**A**) Expression of *MAGEA11* in pan-cancer. (**B**) *MAGEA11* mRNA expression in gastric cancer and normal tissues. (**C**) ROC curve. (**D**) Kaplan–Meier survival curve. (**E**) The subcellular distribution of *MAGEA11*. Green is the target protein staining, red is microtubules staining, and blue is nucleus staining. (* *p* < 0.05, ** *p* < 0.01, and *** *p* < 0.001).

To assess the mRNA expression patterns of *MAGEA11* in different cancer types, as shown in Figure 1A, *MAGEA11* was significantly different in 21 cancers compared to normal tissues. According to this information, *MAGEA11* mRNA expression was abnormal in various cancer types.

To examine the expression of *MAGEA11* mRNA in gastric cancer, we analyzed the *MAGEA11* expression data in TCGA. The *MAGEA11* mRNA expression level in gastric cancer tissues (*n* = 375) was significantly higher than that in normal tissues (*n* = 32), according to unpaired data analysis (Figure 1B). At the same time, we supplemented the GTEx database and the GEO database (GSE54129) for joint analysis and also found that the expression of *MAGEA11* in gastric cancer was significantly higher than that in normal tissues (Supplementary Figures S1 and S2). These outcomes showed that the mRNA expression of *MAGEA11* was upregulated in gastric cancer tissues.

Receiver Operating Characteristic (ROC) curve analysis was carried out to examine the diagnostic utility of *MAGEA11* in differentiating gastric cancer samples from normal tissues. As shown in Figure 1C, ROC curve analysis indicated that the area under curve (AUC) value of *MAGEA11* was 0.667, 95% confidence interval (95%CI = 0.596–0.737). The findings implied that *MAGEA11* may be a promising biomarker to distinguish normal tissue from adenocarcinoma tissue. Subsequently, *MAGEA11* mRNA expression and overall survival (OS) were investigated in gastric cancer patients using the Kaplan–Meier (KM) curve. Figure 1D demonstrates that patients with high-level gastric cancer with *MAGEA11* had shorter OS than patients with low-level gastric cancer with *MAGEA11* (*p* = 0.034). At the same time, we applied the GEO database (GSE84437) to verify the survival and also found that the survival of the *MAGEA11* high expression group was significantly lower than that of the *MAGEA11* low expression group (*p* = 0.02) (Supplementary Figure S3). Finally, we analyzed the expression of *MAGEA11* at subcellular localization, and we found the subcellular distribution of *MAGEA11* mainly localized in the nucleoplasm (Figure 1E).

### 3.2. Independent Risk Factors

**Figure 2:**
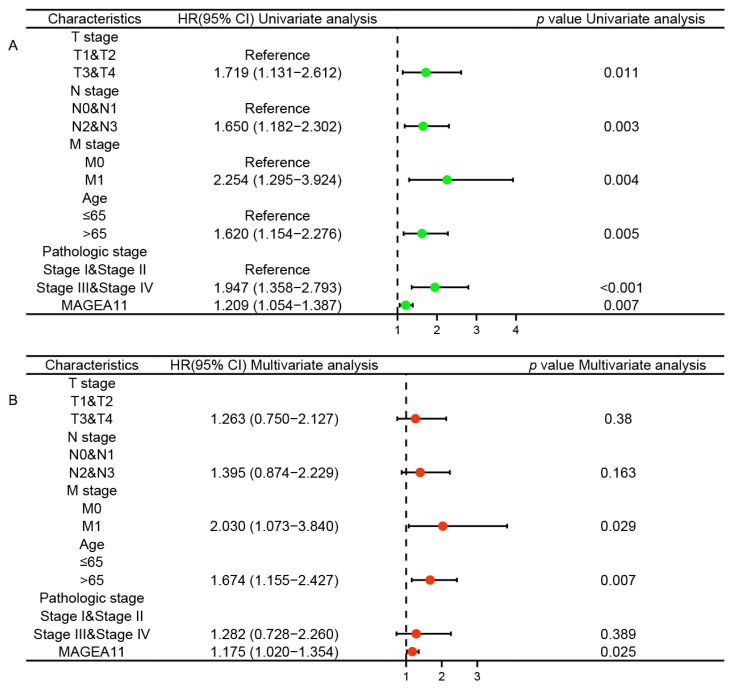
Cox regression analysis of *MAGEA11* expression and related clinical features in overall survival of STAD patients. (**A**) Univariate Cox regression analysis. (**B**) Multivariate Cox regression analysis.

To explore whether *MAGEA11* acts as an independent risk factor in STAD, we performed Cox regression analysis. Univariate Cox regression analysis showed that T stage, N stage, M stage, age, pathologic stage, and *MAGEA11* expression were associated with OS. Furthermore, multivariate Cox regression analysis showed that M stage (HR = 2.030, *p* = 0.029), age (HR = 1.674, *p* = 0.007), and *MAGEA11* expression (HR = 1.175, *p* = 0.025) were independent prognostic factors for STAD patients (Figure 2). These results suggest that in STAD, up-regulated MAGEA11 is associated with poorer prognosis and acts as an independent risk factor.

### 3.3. Gene Enrichment Analysis

**Figure 3:**
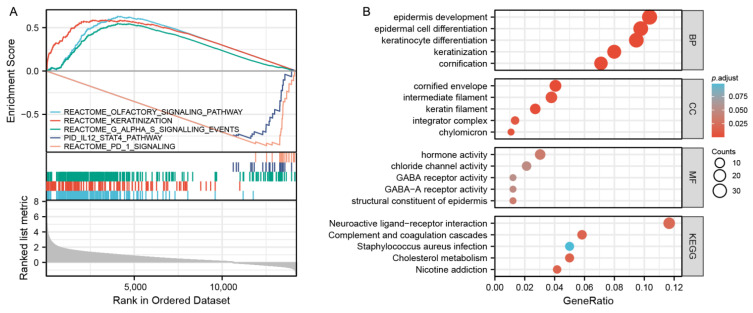
Gene enrichment analysis. (**A**) GSEA functional analysis of genes enriched in representative pathways. (**B**) GO and KEGG analysis of DEGs in *MAGEA11* low and high expression samples.

According to the median expression value of *MAGEA11*, we examined differentially expressed genes (DEGs) between low and high *MAGEA11* expression groups to understand the biological function. A GSEA pathway analysis was performed (Figure 3A). The results show that high expression is enriched in REACTOME_OLFACTORY_SIGNALING_PATHWAY, REACTOME_KERATINIZATION and REACTOME_G_ALPHA_S_SIGNALLING_EVENTS. Low expression was found to be enriched in PID_IL12_STAT4_PATHWAY, REACTOME_PD_1_SIGNALING. Meanwhile, we completed GO and KEGG analysis (Figure 3B). The first five BP terms were associated with cornification, keratinocyte differentiation, keratinization, epidermal cell differentiation, and epidermis development. The first five CC terms are related to cornified envelope, keratin filament, intermediate filament, chylomicron, and integrator complex. The first five MF terms are related to hormone activity, structural constituent of epidermis, GABA-A receptor activity, chloride channel activity, and GABA receptor activity. KEGG is mainly enriched in cholesterol metabolism, complement and coagulation cascades, nicotine addiction, neuroactive ligand–receptor interaction, and Staphylococcus aureus infection.

### 3.4. Construction and Verification of Nomogram

**Figure 4:**
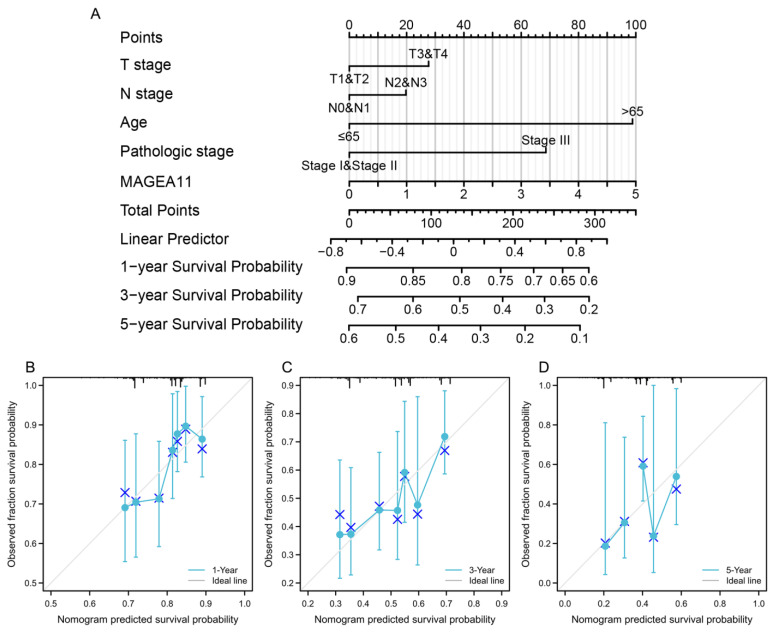
A nomogram was constructed to predict the survival probability of STAD patients. (**A**) Nomogram for predicting 1-, 3-, and 5-year STAD survival probability, including *MAGEA11* and clinical risk factors. (**B**–**D**) Calibration curves of the nomogram predicting OS in patients with STAD. Calibration curves of the nomogram predict 1-year (**B**), 3-year (**C**), and 5-year (**D**) OS in patients with STAD.

First, we constructed a clinical baseline table of STAD patients(Table 1). Subsequently, to provide a quantitative method for predicting the prognosis of STAD patients, a nomogram composed of *MAGEA11* and related clinical risk factors (T/N stage, age, pathological stage) was constructed. A point ruler is used to assign points to these variables in this nomogram, which is based on a Cox analysis. Determine the variable’s point total by drawing a line, and then change the sum of the points assigned to each variable to a range of 0 to 100. The sum of the integrals for each variable is recorded as the final score. The survival probabilities of STAD patients at 1, 3, and 5 years were calculated vertically from the total point axis to the outcome axis (Figure 4A). In order to test the predictive ability of the nomogram, it was evaluated by the calibration curve of the predictive model. The 1-, 3-, and 5-year calibration curves approximated the 45-degree baseline (Figure 4B–D), indicating a high agreement between the actual and expected survival for this model.

### 3.5. PPI Network and Functional Notes

**Figure 5:**
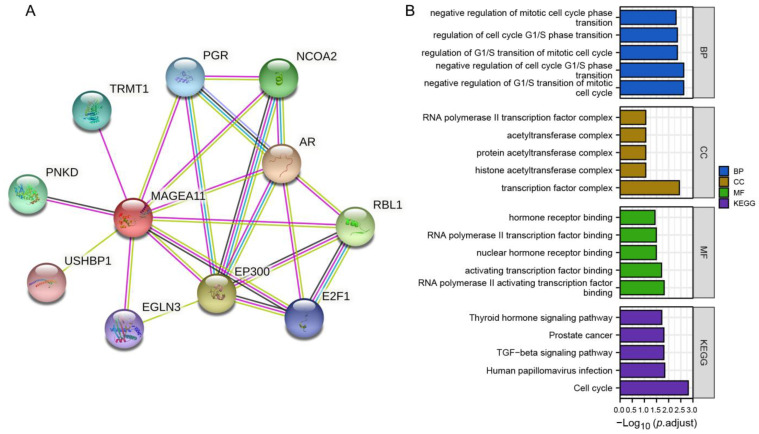
PPI network and feature enrichment analysis. (**A**) *MAGEA11* and its interacting gene network. (**B**) Functional enrichment analysis of interacting genes.

The PPI network was constructed from the STRING database, and GO and KEGG analyses were performed. Figure 5A shows a network of *MAGEA11* and its 10 interacting genes. As shown in Figure 5B, the interacting genes in BP are related to negative regulation of G1/S transition of mitotic cell cycle, negative regulation of cell cycle G1/S phase transition, regulation of G1/S transition of mitotic cell cycle, regulation of cell cycle G1/S phase transition, and negative regulation of mitotic cell cycle phase transition. In CC, interacting genes are mainly enriched in transcription factor complex, histone acetyltransferase complex, protein acetyltransferase complex, acetyltransferase complex, RNA polymerase II transcription factor complex, and other functions. MF annotation shows that these genes are involved in RNA polymerase II activating transcription factor binding, activating transcription factor binding, nuclear hormone receptor binding, RNA polymerase II transcription factor binding, hormone receptor binding, and other functions. In KEGG enrichment analysis, interacting genes were found in cell cycle, human papillomavirus infection, TGF-beta signaling pathway, prostate cancer, and thyroid hormone signaling pathway.

### 3.6. MAGEA11 Mutation Analysis

We observed the genetically altered status of *MAGEA11* in different tumor samples from the TCGA cohort. “Amplification” type of sCNA was the predominant type in gastric cancer cases, which showed an altered frequency of about 2.27% (Supplementary Figure S4A). Supplementary Figure S4B further illustrated the type, locus, and case number of *MAGEA11* genetic alterations. The results suggested that the missense of *MAGEA11* was the main type of genetic alterations. Alterations in the Tudor domain of R235C/L were detected in three different cancer patients, and we observed the R235C/L site in the three-dimensional structure of *MAGEA11* protein (Supplementary Figure S4C). Finally, we observed the distribution of T cell CD4+ memory resting, T cell CD8+, neutrophils, and B cell naive immune infiltration in different sCNA status of *MAGEA11* in the TIMER database (Supplementary Figure S4D–G).

### 3.7. Correlation of MAGEA11 with Immune Checkpoints and Immune Cell Infiltration

**Figure 6:**
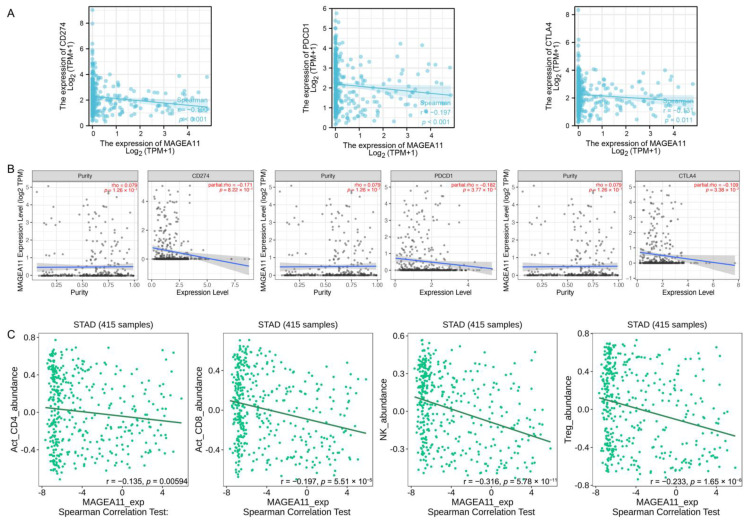
Correlation of *MAGEA11* with immune checkpoints and immune cell infiltration. Correlation between *MAGEA11* and PD-L1 (CD247), PD-1(PDCD-1), CTLA-4 expression in (**A**) the TCGA-STAD database and (**B**) TIMER database, respectively. (**C**) *MAGEA11* and Act_CD4 + T cells, Act_CD8 + T cells, NK cells, and Treg cells’ correlation abundance.

PD-L1(CD274), PD-1(PDCD-1), and CTLA-4 were key immune checkpoints involved in tumor immune escape. Given *MAGEA11*’s putative oncogenic function in STAD, the association of *MAGEA11* with PD-L1, PD-1, and CTLA-4 was investigated in the TCGA-STAD database (Figure 6A) and TIMER databases (Figure 6B). In STAD, there was a substantial negative correlation between *MAGEA11* and PD-L1, PDCD1, and CTLA-4.

In the TISIDB database, we simultaneously evaluated the relationship between *MAGEA11* expression and lymphocytes. As shown in Figure 6C, the expression of *MAGEA11* correlated with lymphocyte abundance. Act_CD4+ T cells (r = −0.135, *p* = 0.00594), Act_CD8+ T cells (r = −0.197, *p* = 5.51 × 10^−5^), NK cells (r = −0.316, *p* = 5.78 × 10^−11^), and Treg cells (r = −0.233, *p* = 1.65 × 10^−6^). These data suggest that high expression of *MAGEA11* inhibits immune cell infiltration, creating an environment for immune escape tolerance.

### 3.8. Drug Sensitivity

**Figure 7:**
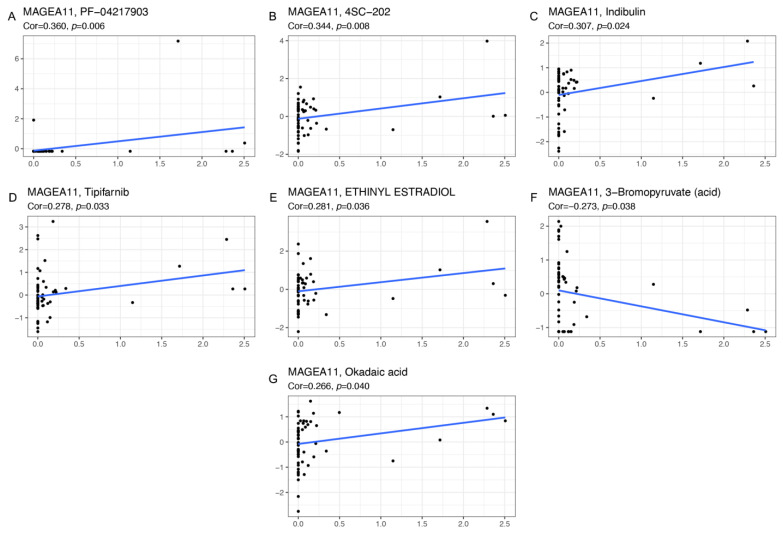
The correlation diagram of gene and drug sensitivity. (**A**) PF-04217903, (**B**) 4SC-202, (**C**) Indibulin, (**D**) Tipifarnib, (**E**) ETHINYL ESTRADIOL, (**F**) 3−Bromopyruvate (acid), and (**G**) Okadaic acid. The x-axis is gene expression, and the y-axis is drug sensitivity.

Genes associated with the sensitivity and resistance of cancer drugs have been extensively studied [32,33]. We explored the sensitivity of *MAGEA11* gene to common antitumor drugs using the CellMiner database, and further calculated the relationship between gene expression and drug sensitivity. The study found that the expression of *MAGEA11* gene was related to 7 kinds of drug sensitivity (Figure 7). Among them, *MAGEA11* was negatively correlated with 3−Bromopyruvate (acid) and positively correlated with PF-04217903, 4SC-202, Indibulin, Tipifarnib, ETHINYL ESTRADIOL, and Okadaic acid.

## 4. Discussion

Gastric cancer seriously threatens people’s health and life, with high incidence, low early diagnosis rate, and low survival rate [34]. Therefore, the identification of diagnostic and prognostic markers in gastric cancer has rapidly become a very important subject area. There are many types of diagnostic and prognostic biomarkers for cancers, including gene expression [35], histopathological images [36,37,38], tissue microbes [39], and tumor microenvironment [40], among which gene expression might be the most studied. At present, there is no clear research report on the role of *MAGEA11* in human gastric cancer, and it is worthy of attention to explore the expression of *MAGEA11* and its effect on gastric cancer.

Studies have shown that *MAGEA11* can be highly expressed in a variety of tumors and may be expected to be a marker for prognosis and diagnosis. Shiheng et al. found that *MAGEA11* can be used as a diagnostic and prognostic marker for head and neck squamous cell carcinoma [41], Shina et al. demonstrated that *MAGEA11* can promote the proliferation of esophageal squamous cell carcinoma [42], and Shifeng also demonstrated that *MAGEA11* can predict renal cell carcinoma risk and survival [19]. In this paper, we found that *MAGEA11* was significantly highly expressed in gastric cancer patients, and it was correlated with the survival of patients. We believe that *MAGEA11* is associated with the poor prognosis of gastric cancer because patients with high expression of *MAGEA11* had relatively shorter survival times. M stage, age, and *MAGEA11* could all be independent prognostic factors, according to additional univariate and multivariate Cox risk regression analysis.

Subsequently, we found that gene enrichment functional analysis mainly focused on genes related to keratinization, and the pathways were mainly enriched in cholesterol metabolism, complement and coagulation cascades, nicotine addiction, neuroactive ligand–receptor interactions, and Staphylococcus aureus infection. The current study found that keratinocytes can be one of the risk factors for gastric cancer [43] and play a large role in the progression of gastric cancer [44], and keratinocytes can specifically stimulate the proliferation of gastric cancer cells [45]. Gastric cancer is also associated with fatty acid metabolism, which can lead to increased rates of fatty acid and cholesterol synthesis, and lipid metabolism has been implicated in gastric cancer progression [46]. Complement levels were significantly elevated in gastric cancer [47], complement activation in the tumor microenvironment enhanced tumor growth and increased metastasis [48], and complement could be used as one of the markers of poor prognosis in gastric cancer [49]. Cancer research has also revealed coagulation-related events during tumor onset, progression, and metastasis [50]; coagulation factors can be abundantly expressed in gastric cancer [51]; and differentially expressed RNAs in gastric cancer are mainly rich in neuroactive ligand–receptor interactions’ effect [52]; nicotine and tobacco addiction can increase risk [53]; there is increasing evidence that bacterial infection is also an important factor in inducing cancer [54]. GABA receptors are highly expressed in gastric cancer [55] and can enhance the proliferation ability of gastric cancer cells [56]. Additionally, we created a nomogram in this article to visually display five clinicopathological variables (*MAGEA11*, age, pathological stage, T/N stage). The calibration curve suggests that the nomogram has good predictive power. Moreover, we analyzed the interaction genes of *MAGEA11* by string and analyzed by enrichment. They were found to be closely related to the cell cycle, transcription, and hormones. These also make preliminary preparations for the follow-up biological mechanism research.

Tumor immune infiltration, tumor microenvironment, immune checkpoint molecules, and immune cell pathways are dynamic and play a role in tumorigenesis and progression [57]. Earlier research [58] has indicated that immune cell infiltration in the tumor microenvironment has prognostic value in a range of cancers. PD-1 is a transmembrane inhibitory protein that is expressed on T cells, B cells, natural killer cells (NK), and myeloid-derived suppressor cells (MDSC) [59]. The PD-1/PD-L1 pathway can form a local immunosuppressive environment [60] and play an important role in gastric cancer cell immune escape [61]. CTLA-4 mediates immunosuppression by indirectly reducing signaling through the costimulatory receptor CD28 [62]. Our results found that *MAGEA11* is negatively correlated with PD-L1, PD-1, and CTLA-4, and it may be that anti-PD-1/PD-L1 has poor efficacy in the high expression group of *MAGEA11*. Through TISIDB database analysis, it was found that *MAGEA11* expression was negatively correlated with CD4+ T cells, CD8+ T cells, NK cells, and Treg cells. Circulating CD4+ T cells can target cancer cell surface antigens and activate peripheral blood CD8+ T cells, allowing them to enter the tumor microenvironment and kill cancer cells [63,64]. Bihui et al. confirmed that NK cells have strong antitumor activity and can effectively eliminate and inhibit gastric cancer cells [65,66]. Our data indicated that with high expression of *MAGEA11*, the immune microenvironment inhibits the infiltration of immune cells, creating an environment for immune escape tolerance. Finally, we explored seven drugs that inhibit *MAGEA11* through the CellMiner database. Preliminary studies support that PF-04217903, 4SC-202, Indibulin, Tipifarnib, ETHINYL ESTRADIOL, and Okadaic acid can target *MAGEA11*, which is expected to make new progress in gastric cancer treatment.

We also need to enrich and validate the above findings using clinical samples and cell animal experiments to better understand the detailed mechanism of *MAGEA11* and gastric cancer immune invasion. Finally, we discovered for the first time in this study that *MAGEA11* is highly upregulated in gastric cancer as a potential prognostic marker and may play a specific role in immune infiltration.

## 5. Conclusions

In the present study, we found for the first time that *MAGEA11* is upregulated and has poor prognosis in gastric cancer and may play a specific role in immune infiltration. Thus, *MAGEA11* can be used as a potential prognostic marker.

## Figures and Tables

**Table 1:** TCGA Clinical Baseline Data Sheet for STAD Patients.

Characteristic	Low Expression of *MAGEA11*	High Expression of *MAGEA11*	*p*
n	187	188	
T stage, n (%)			0.358
T1	7 (1.9%)	12 (3.3%)	
T2	37 (10.1%)	43 (11.7%)	
T3	83 (22.6%)	85 (23.2%)	
T4	56 (15.3%)	44 (12%)	
N stage, n (%)			0.755
N0	57 (16%)	54 (15.1%)	
N1	52 (14.6%)	45 (12.6%)	
N2	36 (10.1%)	39 (10.9%)	
N3	34 (9.5%)	40 (11.2%)	
M stage, n (%)			1.000
M0	163 (45.9%)	167 (47%)	
M1	12 (3.4%)	13 (3.7%)	
Gender, n (%)			0.251
Female	61 (16.3%)	73 (19.5%)	
Male	126 (33.6%)	115 (30.7%)	
Age, median (IQR)	67.5 (57.75, 74)	67 (59, 73)	0.715

## Data Availability

The data used to support the results are available at TCGA (https://portal.gdc.cancer.gov/ (accessed on 10 June 2022)), GTEx (https://www.gtexportal.org/home/index.html (accessed on 10 June 2022)), GEO (https://www.ncbi.nlm.nih.gov/geo/ (accessed on 10 June 2022)), and GDSC (https://www. cancerrxgene.org/ (accessed on 10 June 2022)).

## Supplementary Materials

The following supporting information can be downloaded at: https://www.mdpi.com/article/10.3390/diagnostics12102506/s1. Supplementary Figure S1: The expression of *MAGEA11* in STAD and normal tissues based on TCGA and GTEx databases. (*** *p* < 0.001). Supplementary Figure S2: The expression of *MAGEA11* in STAD and normal tissues based on GEO databases. (* *p* < 0.05). Supplementary Figure S3: Survival analysis of *MAGEA11* expression in STAD based on GEO database. Supplementary Figure S4: *MAGEA11* mutation analysis. (A)*MAGEA11* gene mutation signature in different tumors of TCGA. (B) The mutation type and the mutation frequency of the mutation site. (C) The mutation site (R235C/L) with the highest mutation frequency in the three-dimensional structure of *MAGEA11*. (D-G) T cell CD4+ memory resting, T cell CD8+, B cell naive, and Neutrophil immune infiltration distribution between different sCNA status of *MAGEA11*.

## References

[1] Sung H., Ferlay J., Siegel R.L., Laversanne M., Soerjomataram I., Jemal A., Bray F. (2021). Global Cancer Statistics 2020: GLOBOCAN Estimates of Incidence and Mortality Worldwide for 36 Cancers in 185 Countries. CA Cancer J. Clin..

[2] Ilic M., Ilic I. (2022). Epidemiology of stomach cancer. World J. Gastroenterol..

[3] Hu J., Yang Y., Ma Y., Ning Y., Chen G., Liu Y. (2022). Survival benefits from neoadjuvant treatment in gastric cancer: A systematic review and meta-analysis. Syst. Rev..

[4] Chen J.Y., Lin G.T., Chen Q.Y., Zhong Q., Liu Z.Y., Que S.J., Wang J.B., Lin J.X., Lu J., Cao L.L. Textbook outcome, chemotherapy compliance, and prognosis after radical gastrectomy for gastric cancer: A large sample analysis. Eur. J. Surg. Oncol..

[5] Zang X., Jiang J., Gu J., Chen Y., Wang M., Zhang Y., Fu M., Shi H., Cai H., Qian H. (2022). Circular RNA EIF4G3 suppresses gastric cancer progression through inhibition of beta-catenin by promoting delta-catenin ubiquitin degradation and upregulating SIK1. Mol. Cancer.

[6] Wang Y., Jia Z., Gao J., Zhou T., Zhang X., Zu G. (2022). Clinicopathological and Prognostic Value of USP22 Expression in Gastric Cancer: A Systematic Review and Meta-Analysis and Database Validation. Front. Surg..

[7] Liu H., Qiu C., Wang B., Bing P., Tian G., Zhang X., Ma J., He B., Yang J. (2021). Evaluating DNA Methylation, Gene Expression, Somatic Mutation, and Their Combinations in Inferring Tumor Tissue-of-Origin. Front. Cell Dev. Biol..

[8] He B., Lang J., Wang B., Liu X., Lu Q., He J., Gao W., Bing P., Tian G., Yang J. (2020). TOOme: A Novel Computational Framework to Infer Cancer Tissue-of-Origin by Integrating Both Gene Mutation and Expression. Front. Bioeng. Biotechnol..

[9] Yamaji T. (1984). Antidiuretic hormone and its disorders. Horumon Rinsho.

[10] Peikert T., Specks U., Farver C., Erzurum S.C., Comhair S.A. (2006). Melanoma antigen A4 is expressed in non-small cell lung cancers and promotes apoptosis. Cancer Res..

[11] Suyama T., Ohashi H., Nagai H., Hatano S., Asano H., Murate T., Saito H., Kinoshita T. (2002). The MAGE-A1 gene expression is not determined solely by methylation status of the promoter region in hematological malignancies. Leuk. Res..

[12] Su S., Minges J.T., Grossman G., Blackwelder A.J., Mohler J.L., Wilson E.M. (2013). Proto-oncogene activity of melanoma antigen-A11 (MAGE-A11) regulates retinoblastoma-related p107 and E2F1 proteins. J. Biol. Chem..

[13] Sang M., Lian Y., Zhou X., Shan B. (2011). MAGE-A family: Attractive targets for cancer immunotherapy. Vaccine.

[14] Otte M., Zafrakas M., Riethdorf L., Pichlmeier U., Loning T., Janicke F., Pantel K. (2001). MAGE-A gene expression pattern in primary breast cancer. Cancer Res..

[15] Lin J., Lin L., Thomas D.G., Greenson J.K., Giordano T.J., Robinson G.S., Barve R.A., Weishaar F.A., Taylor J.M., Orringer M.B. (2004). Melanoma-associated antigens in esophageal adenocarcinoma: Identification of novel MAGE-A10 splice variants. Clin. Cancer Res..

[16] Jang S.J., Soria J.C., Wang L., Hassan K.A., Morice R.C., Walsh G.L., Hong W.K., Mao L. (2001). Activation of melanoma antigen tumor antigens occurs early in lung carcinogenesis. Cancer Res..

[17] Brasseur F., Rimoldi D., Lienard D., Lethe B., Carrel S., Arienti F., Suter L., Vanwijck R., Bourlond A., Humblet Y. (1995). Expression of MAGE genes in primary and metastatic cutaneous melanoma. Int. J. Cancer.

[18] Bergeron A., Picard V., LaRue H., Harel F., Hovington H., Lacombe L., Fradet Y. (2009). High frequency of MAGE-A4 and MAGE-A9 expression in high-risk bladder cancer. Int. J. Cancer.

[19] Su S., Gu Q., Xu A., Shen S., Liu S., Zhang C., Miao C., Qin C., Liu B., Wang Z. (2019). Genetic variations in MAGE-A11 predict the risk and survival of renal cell cancer. J. Cancer.

[20] Zhang W., Hu X., Chakravarty H., Yang Z., Tam K.Y. (2018). Identification of Novel Pyruvate Dehydrogenase Kinase 1 (PDK1) Inhibitors by Kinase Activity-Based High-Throughput Screening for Anticancer Therapeutics. ACS Comb. Sci..

[21] Bai S., Wilson E.M. (2008). Epidermal-growth-factor-dependent phosphorylation and ubiquitinylation of MAGE-11 regulates its interaction with the androgen receptor. Mol. Cell. Biol..

[22] Su S., Blackwelder A.J., Grossman G., Minges J.T., Yuan L., Young S.L., Wilson E.M. (2012). Primate-specific melanoma antigen-A11 regulates isoform-specific human progesterone receptor-B transactivation. J. Biol. Chem..

[23] Askew E.B., Bai S., Blackwelder A.J., Wilson E.M. (2010). Transcriptional synergy between melanoma antigen gene protein-A11 (MAGE-11) and p300 in androgen receptor signaling. J. Biol. Chem..

[24] Askew E.B., Bai S., Hnat A.T., Minges J.T., Wilson E.M. (2009). Melanoma antigen gene protein-A11 (MAGE-11) F-box links the androgen receptor NH2-terminal transactivation domain to p160 coactivators. J. Biol. Chem..

[25] Tomczak K., Czerwinska P., Wiznerowicz M. (2015). The Cancer Genome Atlas (TCGA): An immeasurable source of knowledge. Contemp. Oncol..

[26] Yu G., Wang L.G., Han Y., He Q.Y. (2012). Clusterprofiler: An R package for comparing biological themes among gene clusters. OMICS.

[27] Liu J., Lichtenberg T., Hoadley K.A., Poisson L.M., Lazar A.J., Cherniack A.D., Kovatich A.J., Benz C.C., Levine D.A., Lee A.V. (2018). An Integrated TCGA Pan-Cancer Clinical Data Resource to Drive High-Quality Survival Outcome Analytics. Cell.

[28] Szklarczyk D., Franceschini A., Kuhn M., Simonovic M., Roth A., Minguez P., Doerks T., Stark M., Muller J., Bork P. (2011). The STRING database in 2011: Functional interaction networks of proteins, globally integrated and scored. Nucleic Acids Res..

[29] Bashiri H., Rahmani H., Bashiri V., Modos D., Bender A. (2020). EMDIP: An Entropy Measure to Discover Important Proteins in PPI networks. Comput. Biol. Med..

[30] Zhang Z., Chai H., Wang Y., Pan Z., Yang Y. (2022). Cancer survival prognosis with Deep Bayesian Perturbation Cox Network. Comput. Biol. Med..

[31] Robin X., Turck N., Hainard A., Tiberti N., Lisacek F., Sanchez J.C., Muller M. (2011). pROC: An open-source package for R and S+ to analyze and compare ROC curves. BMC Bioinform..

[32] Liu C., Wei D., Xiang J., Ren F., Huang L., Lang J., Tian G., Li Y., Yang J. (2020). An Improved Anticancer Drug-Response Prediction Based on an Ensemble Method Integrating Matrix Completion and Ridge Regression. Mol. Ther. Nucleic Acids.

[33] Liu X., Yang J., Zhang Y., Fang Y., Wang F., Wang J., Zheng X., Yang J. (2016). A systematic study on drug-response associated genes using baseline gene expressions of the Cancer Cell Line Encyclopedia. Sci. Rep..

[34] Ferlay J., Soerjomataram I., Dikshit R., Eser S., Mathers C., Rebelo M., Parkin D.M., Forman D., Bray F. (2015). Cancer incidence and mortality worldwide: Sources, methods and major patterns in GLOBOCAN 2012. Int. J. Cancer.

[35] Wang B., Yang H., Zhang Y., Tian G., Yang J. (2022). A computational framework to trace tumor tissue-of-origin of 19 cancer types based on RNA sequencing. Res. Sq..

[36] Yang J., Ju J., Guo L., Ji B., Shi S., Yang Z., Gao S., Yuan X., Tian G., Liang Y. (2022). Prediction of HER2-positive breast cancer recurrence and metastasis risk from histopathological images and clinical information via multimodal deep learning. Comput. Struct. Biotechnol. J..

[37] Ye Z., Zhang Y., Liang Y., Lang J., Zhang X., Zang G., Yuan D., Tian G., Xiao M., Yang J. (2022). Cervical Cancer Metastasis and Recurrence Risk Prediction Based on Deep Convolutional Neural Network. Curr. Bioinform..

[38] Liu X., Yuan P., Li R., Zhang D., An J., Ju J., Liu C., Ren F., Hou R., Li Y. (2022). Predicting breast cancer recurrence and metastasis risk by integrating color and texture features of histopathological images and machine learning technologies. Comput. Biol. Med..

[39] Yang M., Yang H., Ji L., Hu X., Tian G., Wang B., Yang J. (2022). A multi-omics machine learning framework in predicting the survival of colorectal cancer patients. Comput. Biol. Med..

[40] Liu J., Lan Y., Tian G., Yang J. (2022). A Systematic Framework for Identifying Prognostic Genes in the Tumor Microenvironment of Colon Cancer. Front. Oncol..

[41] Jia S., Zhang M., Li Y., Zhang L., Dai W. (2020). MAGE-A11 Expression Predicts Patient Prognosis in Head and Neck Squamous Cell Carcinoma. Cancer Manag. Res..

[42] Liu S., Liu F., Huang W., Gu L., Meng L., Ju Y., Wu Y., Li J., Liu L., Sang M. (2018). MAGE-A11 is activated through TFCP2/ZEB1 binding sites de-methylation as well as histone modification and facilitates ESCC tumor growth. Oncotarget.

[43] Tani H., Saito N., Kobayashi M., Kameoka S. (2013). Clinical significance of keratinocyte growth factor and K-sam gene expression in gastric cancer. Mol. Med. Rep..

[44] Yashiro M., Chung Y.S., Kubo T., Hato F., Sowa M. (1996). Differential responses of scirrhous and well-differentiated gastric cancer cells to orthotopic fibroblasts. Br. J. Cancer.

[45] Nakazawa K., Yashiro M., Hirakawa K. (2003). Keratinocyte growth factor produced by gastric fibroblasts specifically stimulates proliferation of cancer cells from scirrhous gastric carcinoma. Cancer Res..

[46] Yu L., Lai Q., Feng Q., Li Y., Feng J., Xu B. (2021). Serum Metabolic Profiling Analysis of Chronic Gastritis and Gastric Cancer by Untargeted Metabolomics. Front. Oncol..

[47] Yuan K., Ye J., Liu Z., Ren Y., He W., Xu J., He Y., Yuan Y. (2020). Complement C3 overexpression activates JAK2/STAT3 pathway and correlates with gastric cancer progression. J. Exp. Clin. Cancer Res..

[48] Afshar-Kharghan V. (2017). The role of the complement system in cancer. J. Clin. Investig..

[49] Bao D., Zhang C., Li L., Wang H., Li Q., Ni L., Lin Y., Huang R., Yang Z., Zhang Y. (2020). Integrative Analysis of Complement System to Prognosis and Immune Infiltrating in Colon Cancer and Gastric Cancer. Front. Oncol..

[50] Repetto O., de Re V. (2017). Coagulation and fibrinolysis in gastric cancer. Ann. N. Y. Acad. Sci..

[51] Takashima H., Koga Y., Manabe S., Ohnuki K., Tsumura R., Anzai T., Iwata N., Wang Y., Yokokita T., Komori Y. (2021). Radioimmunotherapy with an (211) At-labeled anti-tissue factor antibody protected by sodium ascorbate. Cancer Sci..

[52] Guo Z., Liang E., Zhang T., Xu M., Jiang X., Zhi F. (2021). Identification and Validation of a Potent Multi-lncRNA Molecular Model for Predicting Gastric Cancer Prognosis. Front. Genet..

[53] Lund I., Scheffels J. (2014). Perceptions of relative risk of disease and addiction from cigarettes and snus. Psychol. Addict. Behav..

[54] Sheweita S.A., Alsamghan A.S. (2020). Molecular Mechanisms Contributing Bacterial Infections to the Incidence of Various Types of Cancer. Mediat. Inflamm..

[55] Juvale I.I.A., Hassan Z., Has A.T.C. (2021). The Emerging Roles of pi Subunit-Containing GABAA Receptors in Different Cancers. Int. J. Med. Sci..

[56] Maemura K., Shiraishi N., Sakagami K., Kawakami K., Inoue T., Murano M., Watanabe M., Otsuki Y. (2009). Proliferative effects of gamma-aminobutyric acid on the gastric cancer cell line are associated with extracellular signal-regulated kinase 1/2 activation. J. Gastroenterol. Hepatol..

[57] Zeng D., Ye Z., Wu J., Zhou R., Fan X., Wang G., Huang Y., Wu J., Sun H., Wang M. (2020). Macrophage correlates with immunophenotype and predicts anti-PD-L1 response of urothelial cancer. Theranostics.

[58] Engelhard V.H., Rodriguez A.B., Mauldin I.S., Woods A.N., Peske J.D., Slingluff C.L. (2018). Immune Cell Infiltration and Tertiary Lymphoid Structures as Determinants of Antitumor Immunity. J. Immunol..

[59] Vaddepally R.K., Kharel P., Pandey R., Garje R., Chandra A.B. (2020). Review of Indications of FDA-Approved Immune Checkpoint Inhibitors per NCCN Guidelines with the Level of Evidence. Cancers.

[60] Hiramatsu S., Tanaka H., Nishimura J., Yamakoshi Y., Sakimura C., Tamura T., Toyokawa T., Muguruma K., Yashiro M., Hirakawa K. (2020). Gastric cancer cells alter the immunosuppressive function of neutrophils. Oncol. Rep..

[61] Erdogdu I.H. (2019). MHC Class 1 and PDL-1 Status of Primary Tumor and Lymph Node Metastatic Tumor Tissue in Gastric Cancers. Gastroenterol. Res. Pract..

[62] Kennedy A., Waters E., Rowshanravan B., Hinze C., Williams C., Janman D., Fox T.A., Booth C., Pesenacker A.M., Halliday N. (2022). Differences in CD80 and CD86 transendocytosis reveal CD86 as a key target for CTLA-4 immune regulation. Nat. Immunol..

[63] Zhou L., Chong M.M., Littman D.R. (2009). Plasticity of CD4+ T cell lineage differentiation. Immunity.

[64] Zander R., Schauder D., Xin G., Nguyen C., Wu X., Zajac A., Cui W. (2019). CD4(+) T Cell Help Is Required for the Formation of a Cytolytic CD8(+) T Cell Subset that Protects against Chronic Infection and Cancer. Immunity.

[65] Cao B., Liu M., Huang J., Zhou J., Li J., Lian H., Huang W., Guo Y., Yang S., Lin L. (2021). Development of mesothelin-specific CAR NK-92 cells for the treatment of gastric cancer. Int. J. Biol. Sci..

[66] Abdolahi S., Ghazvinian Z., Muhammadnejad S., Ahmadvand M., Aghdaei H.A., Ebrahimi-Barough S., Ai J., Zali M.R., Verdi J., Baghaei K. (2021). Adaptive NK Cell Therapy Modulated by Anti-PD-1 Antibody in Gastric Cancer Model. Front. Pharmacol..

