# Supplementary figures and images for: MAGEA11 as a STAD Prognostic Biomarker Associated with Immune Infiltration
Source: Diagnostics (Basel). 2022 Oct 16;12(10):2506. doi: 10.3390/diagnostics12102506 (PMC9600629; doi:10.3390/diagnostics12102506)

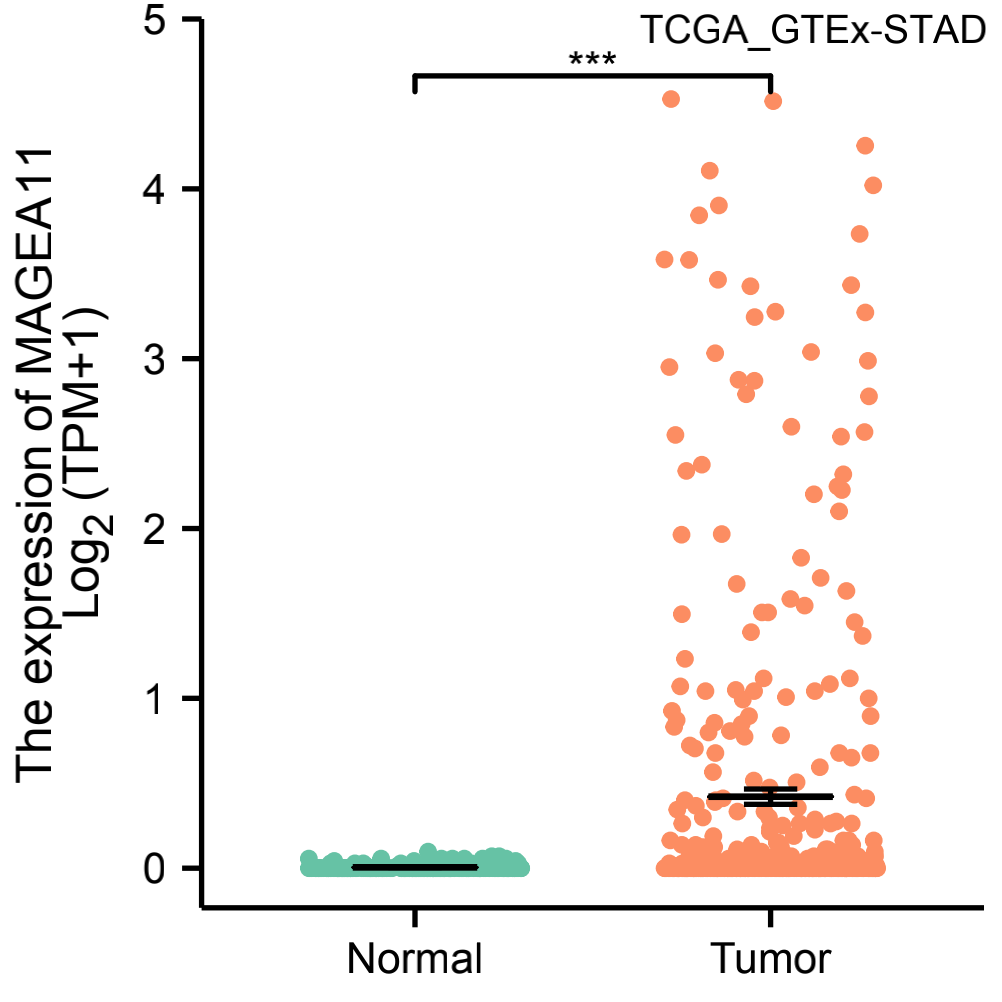

Supplement: Supplementary file 1 [file diagnostics-12-02506-s001.zip › Supplementary Figure S1.tif]

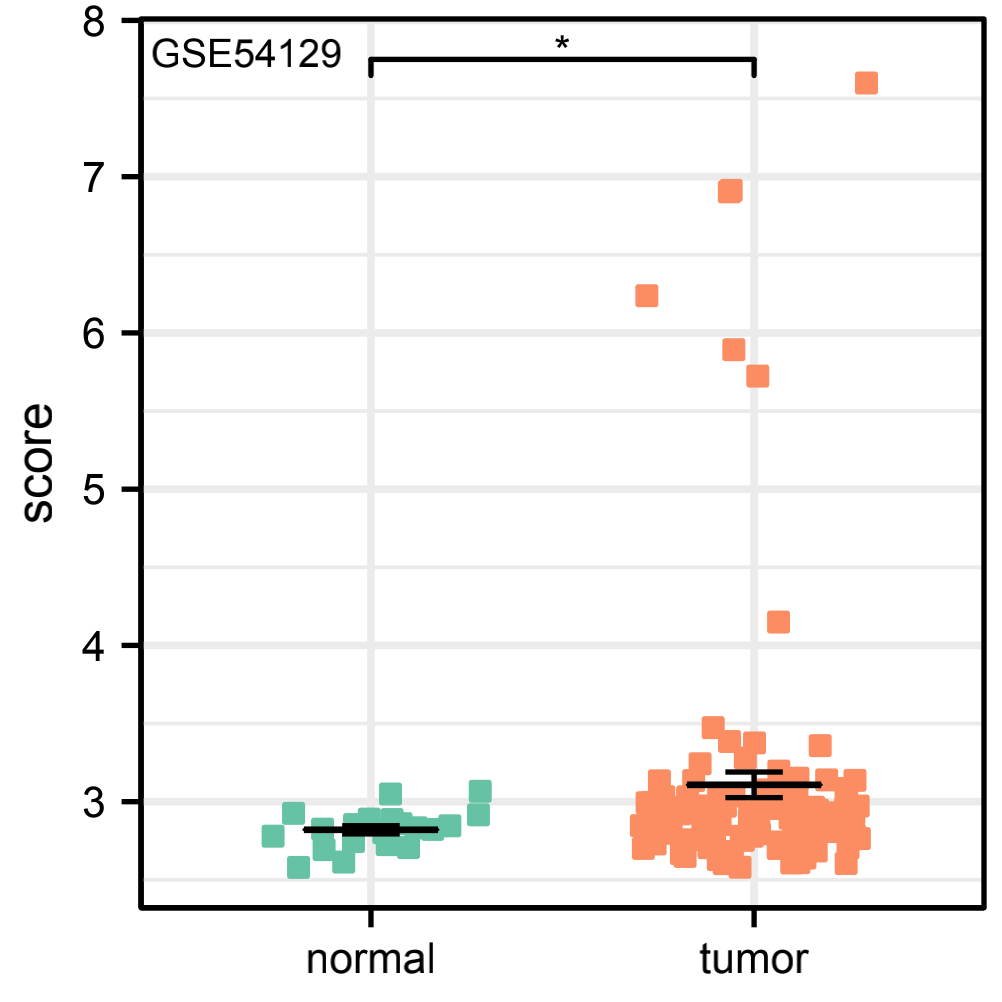

Supplement: Supplementary file 1 [file diagnostics-12-02506-s001.zip › Supplementary Figure S2.tif]

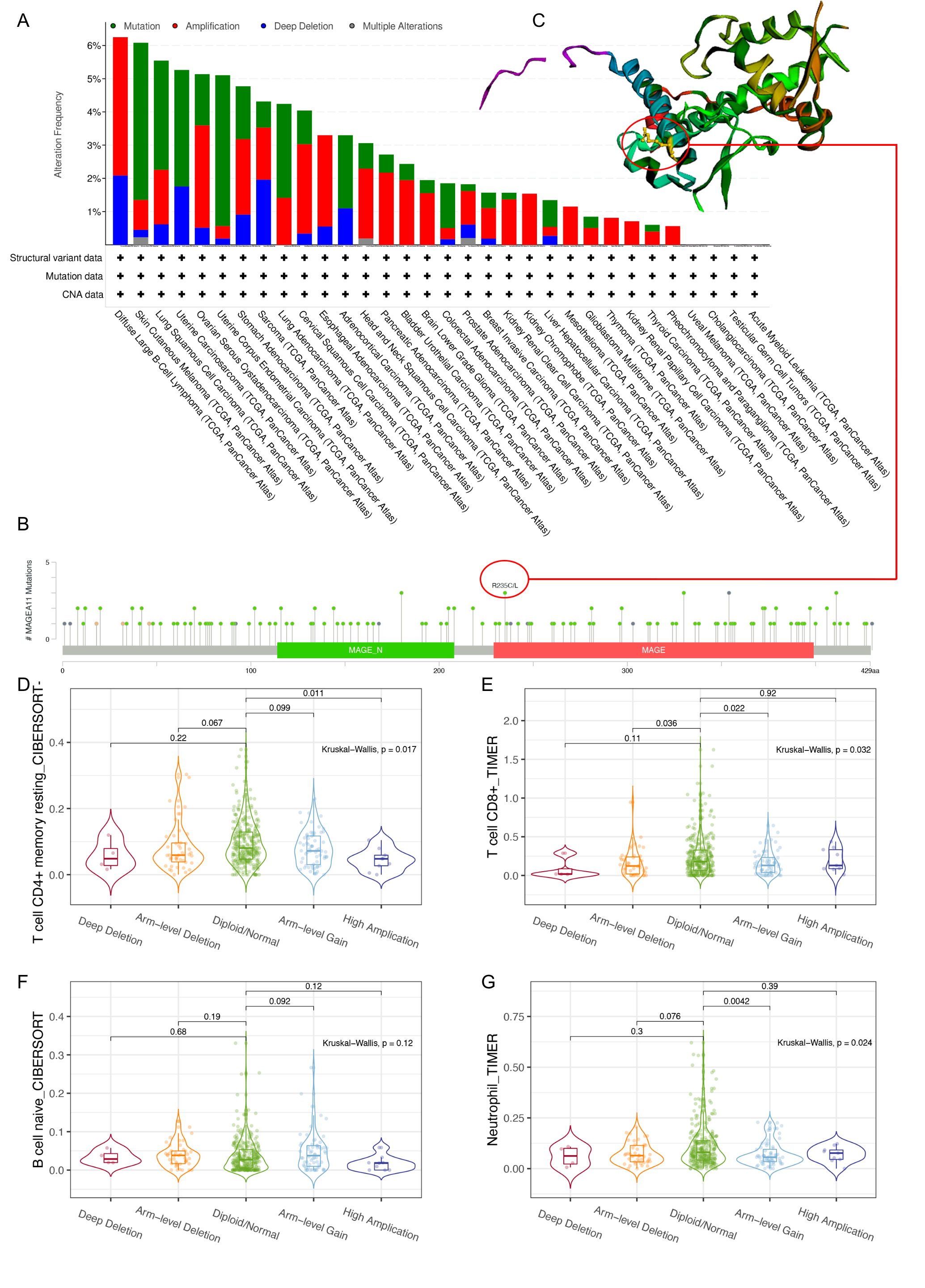

Supplement: Supplementary file 1 [file diagnostics-12-02506-s001.zip › Supplementary Figure S4.tif]
